# Targeting family functioning, acculturative stress, and sugar-sweetened beverage consumption for obesity prevention: findings from the Hispanic community children’s health study/study of Latino youth

**DOI:** 10.1186/s12889-020-09658-6

**Published:** 2020-10-14

**Authors:** Roger Figueroa, Carmen R. Isasi, Krista M. Perreira, Amanda C. McClain, Linda C. Gallo, Daniela Sotres-Alvarez, Alan M. Delamater, Martha Daviglus, Linda Van Horn, Josiemer Mattei

**Affiliations:** 1 Division of Nutritional Sciences, College of Human Ecology, Cornell University, Ithaca, NY USA; 2 Department of Epidemiology and Population Health, Albert Einstein College of Medicine, New York, NY USA; 3 Department of Social Medicine, University of North Carolina School of Medicine, Chapel Hill, NC USA; 4 School of Exercise and Nutritional Sciences, San Diego State University, San Diego, CA USA; 5 Department of Psychology, San Diego State University, San Diego, CA USA; 6 Department of Biostatistics, Gillings School of Global Health, University of North Carolina, Chapel Hill, NC USA; 7 Department of Pediatrics, Mailman Center for Child Development, University of Miami, Miami, FL USA; 8 Department of Preventive Medicine, Feinberg School of Medicine, Northwestern University, Chicago, IL USA; 9 Department of Nutrition, Harvard T.H. Chan School of Public Health, Harvard University, Boston, MA USA

**Keywords:** Hispanic/Latino, Acculturation, Adolescence, Sugar-sweetened beverages (SSB) consumption, Family functioning

## Abstract

**Background:**

Maintaining a bond with one’s family as well coping with stress while acculturating to the US may protect Hispanic/Latino youth from increased sugar-sweetened beverages (SSB) consumption, which heightens the risk for overweight and obesity. This study aims to examine associations between acculturative stress, family functioning, and SSB consumption by acculturation status among U.S. Hispanic/Latino youth.

**Methods:**

With cross-sectional data on 1465 youth 8-16y (49.6% females) participating in the Hispanic Community Children’s Health Study/Study of Latino Youth, we classified youths into four acculturation groups – assimilated, integrated, marginalized/separated, and unclassified. SSB consumption was assessed through two 24-h diet recalls and defined as intake frequency of soda, fruit juice, sweetened soft and fruit drinks. Multi-group path regression models were used to test associations of Hispanic/Latino youth’ acculturative stress and family functioning with SSB consumption, as well as the moderating role of acculturation status.

**Results:**

When controlling for age, sex, and study site, acculturative stress (*β* = − 0.13, *p =* 0.01) was inversely associated with SSB, and poor family functioning (*β* = 0.11, *p =* 0.07) was only marginally associated with SSB consumption among youth classified as assimilated but not among youth classified as integrated, marginalized/separated, or unclassified.

**Conclusions:**

A socio-ecological perspective that incorporates the role of key acculturation-related factors across multiple levels may aid efforts to identify mechanisms that influence the relationship between acculturation status and diet among Hispanic/Latino youth and their families.

## Background

Global consumption of added sugars are the main source of empty calories contributing to daily energy intake, obesity, and diabetes [1–9], which is alarmingly prevalent in children’s and youth’ diets [10]. A popular form of added sugar intake in this age group, sugar-sweetened beverages (SSB), substantially contributes to daily energy intake [10]. SSB include soft drinks (i.e., soda, fruit punches, lemonades), fruit juices and drinks, sports drinks, as well as energy drinks [11]. The major source of added sugar from SSB is 100% fruit juice, while both 100% fruit juice and soft drinks contribute similarly to total energy intake [12]. Large amounts of added sugars in SSB may also increase the risk of obesity by contributing to a high glycemic load and exacerbating insulin response [13]. SSB consumption, along with the daily calories sold per capita per day from all SSB, is highest in both North and Latin America [4], and is prevalent among Hispanic/Latino children and youth in Latin America and the United States (U.S.) [14, 15]. These numbers have pronounced implications for native and immigrant youth of Hispanic/Latino heritage living in the U.S., as they are disproportionally affected by obesity [16].

Existing disparities in SSB consumption and health outcomes such as obesity among Hispanic/Latino youth and their families living in the US may be associated with adoption of US customs and behaviors, versus maintenance of Latin American customs and behaviors [17]. Acculturation, broadly defined as the multidimensional process through which individuals economically, psychologically, and socio-culturally adapt from one cultural context to another due to migration or other life experiences [18, 19], has been associated with dietary behaviors and other obesity-related behaviors among Hispanic/Latino youth [20]. Research suggests that greater language-based acculturation to the U.S. among Hispanic/Latino families is associated with negative health outcomes and less healthful dietary behaviors [17], including greater SSB consumption than their U.S.-born non-Hispanic white counterparts [21]. Recently, Arandia et al. (2018) examined associations between a multi-dimensional measure of acculturation, the Acculturation Habits, and Interests Multicultural Scale for Adolescents (AHIMSA), and Hispanic/Latino youth’s eating behaviors [22]. They found no differences in consumption of empty calories by acculturation group [22]. However, the study did not specifically examine SSB consumption [23].

Because acculturation tends to occur more rapidly among Hispanic/Latino youth than among their parents [24], understanding the role of acculturation on SSB consumption patterns in youth is essential. One plausible factor influencing SSB consumption through acculturation is acculturative stress. Acculturative stress can be defined as the psychological impact of adaptation to a new culture [25]. For Hispanics who move to the U.S., there are a number of significant stressors (i.e., language conflict, family acculturation conflicts, perceived discrimination) that can be pervasive, intense, and lifelong due to acculturation. For example, rapid acculturation to the US among youth may lead to the adoption of unhealthy behaviors through increasing inter-generational stress within families (i.e., family acculturation conflicts), and subsequent loss of Hispanic/Latino customs and behaviors [26]. Thus, acculturative stress may play a significant role influencing health outcomes among Hispanic/Latino families.

The Hispanic/Latino family is a vital source of support, comprised of interdependent relationships that may cultivate cultural values and discourage family dysfunction [27, 28]. This is important as youth in more dysfunctional families engage in unhealthier behaviors [29]. However, the role of family functioning in influencing SSB consumption, moderated by acculturation, remains an untested hypothesis.

This study aimed to examine the associations of acculturative stress and family functioning with SSB consumption by acculturation status among U.S. Hispanic/Latino youth. We examined such associations under the assumption that on average, youth experience more rapid acculturation attainment, as well as youth adopt certain acculturation statuses on the basis of characteristics and experiences that are heterogenous across groups. We hypothesized that Hispanic/Latino youths’ SSB consumption would differ by acculturation status and that acculturative stress and family functioning would be associated with Hispanic/Latino youth’ SSB consumption across acculturation statuses.

## Methods

### Data source and study sample

This study used data from Hispanic Community Children’s Health Study/Study of Latino Youth (SOL Youth) ancillary study to the Hispanic Community Health Study/Study of Latinos (HCHS/SOL) study, which is a comprehensive longitudinal multicenter community–based cohort study of Hispanic/Latino adults in the United States [30]. Between 2008 and 2011, 16,415 U.S. Hispanic/Latino adults were recruited from a random sample of households in four communities (i.e., Bronx, NY; Chicago, IL; Miami, FL; and San Diego, CA).

Between 2012 and 2014, SOL Youth recruited 1465 youth aged 8–16 years whose parent or caregiver was part of HCHS/SOL [31, 32]. For these analyses, we included youth with complete and valid SSB consumption data, as well as complete and valid data for family functioning, socio-cultural factors, and demographics. Study questionnaires to collect these data were administered by bilingual staff and in the youth language of preference. We excluded 210 cases due to incomplete data across key study variables. The overarching goal of the SOL-Youth study was to evaluate the influence of youth acculturation, effects of intergenerational influence in acculturation between youth and parents, parenting practices, as well as psychosocial functioning on cardiometabolic risk in Hispanic/Latino youth [31, 32]. SOL-Youth had a cross-sectional design and included children who were assented to participate through voluntarily written informed consent. The study was conducted with approval of the Institutional Review Board at all study institutions, including the HCHS/SOL coordinating center and laboratory.

### Dependent measures

#### SSB consumption

SSB consumption was measured through two 24-h dietary recalls from each child, with parental assistance if necessary. The initial dietary recall was conducted in person and the second dietary recall was conducted by telephone within a month of the first assessment. The Nutrition Data System for Research (NDSR) software was used to collect the data and to categorize SSB consumed into the following groups: (a) sugar sweetened beverages; (b) 100% fruit juice; (c) sweetened soft drinks; and (d) sweetened fruit drinks. The average frequency of servings per day (8 fluid ounces) across beverage categories was used as the outcome measure.

### Independent measures

#### Acculturation

Hispanic/Latino youth’ acculturation status was measured using the Acculturation, Habits, and Interests Multicultural Scale for Adolescents (AHIMSA) [33]. Scale scores ranged from 1 to 8. Under the assumption that acculturation itself is a dynamic process and that AHIMSA contains correlated sub-scales, youth were grouped into categories based on their acculturation status: Both Countries Orientation (Integration), United States orientation (Assimilation), orientation to other or neither country (Marginalization or Separation), and Unclassified. This categorical variable was used as grouping variable for descriptive purposes as well as the moderator in the final analytical output.

#### Acculturative stress

Hispanic/Latino youth’ acculturative stress was measured using the Acculturative Stress Index [34] through three indicators: language conflict, family acculturation conflicts, and discrimination. To denote acculturative stress in our study, we developed a latent construct of acculturative stress composed of the three indicators aforementioned. This approach was used given that it accounts for measurement error, and tests whether these sub-domains represent well the higher construct (acculturative stress). Each indicator used a 5-point scale and, collectively, showed good compatibility to denote acculturative stress as a latent construct (*a* > 0.61; *r* > 0.32; *b* > 0.41).

#### Family functioning

Hispanic/Latino youths’ family functioning was measured using the 12-item General Functioning subscale of the McMaster Family Assessment Device, which measures overall health of the family [35]. Example of statements include: “Planning family activities is difficult because we misunderstand each other;” “We cannot talk to each other about the sadness we feel;” “Making decisions is a problem for our family.” To denote family functioning in our study, the average of all of the items was used. Family functioning scores ranged from 1 to 4, with greater scores indicating poorer family functioning.

#### Covariates

Demographic characteristics included as covariates in all models were youth age in years (8–16 years old), youth’ sex (male or female), birthplace (mainland U.S. or outside U.S.), parental educational attainment (less than high school, high School/GED, four-year college or university or more), family income (less than $20,000, $20,000–$50,000, greater than $50,000), and household composition (continuous). The Healthy Eating Index (HEI-2010) was also used as a covariate to represent overall diet quality [36].

### Statistical analysis

We used descriptive statistics to summarize demographic characteristics and key study variables (dependent and independent variables). Multi-group path regression models were used to test our study hypotheses using Structural Equation Modelling (SEM). Hispanic/Latino youth’ SSB consumption was used as the dependent variable in all models. Hispanic/Latino youth’ acculturative stress and family functioning were used as independent variables on SSB consumption to test our main hypothesis. Hispanic/Latino youth’ acculturation status was used as the categorical grouping variable. Hispanic/Latino youth’ age and sex were used as covariates in all models. We used 0.05 as the significance level. Model fit was assessed using the root mean square error of approximation (RMSEA), standardized root mean square residual (SRMR), and the Comparative Fit Index (CFI) [33]. In order for our models to attain good fit to the data, the following criteria had to be met: RMSEA (≤0.08), SRMR (≤0.10), and CFI (≥0.90) [37]. Missing data was handled using listwise deletion. Statistical software STATA 14 was used to perform all analyses while accounting for the complex survey design, sampling weights, and clustered nature of the data.

## Results

Demographic characteristics are summarized in Table 1. The majority of children were 8–12 years old (57.5%), and males and females were equally represented. A vast majority were born in the mainland United States (77.5%), and half had low family income (52.9%). Most youth were categorized as integrated (46.0%), followed by assimilated (42.5%), and lastly marginalized or separated (11.3%). Overall, youth consumed approximately one SSB per day (0.96 servings/day), although marginalized or separated youth consumed slightly less SSB compared to youth with a stronger U.S. orientation (not statistically significant). Furthermore, fruit juice was the greatest contributor to overall SSB consumption across groups. Prior to testing the path regression models, ANOVA tests indicated mean SSB intake was statistically different across acculturation groups (*F* (2,1301) = 3.43, *p* = 0.04) in unadjusted models. Chi-square tests also indicated statistical differences across acculturation groups in family functioning (*X*^2^ = 15.30, *p* < 0.01).

**Table 1:** Summary of characteristics of Hispanic/Latino youth, *n* = 1465

	Total Sample (*n* = 1465)	^a^Integrated (*n* = 674)	^b^Assimilated (*n* = 495)	^c^Separated or marginalized (*n* = 92)	^d^Unclassified (*n* = 204)
**SSB consumption (servings/day)**	0.96 (0.75)	0.98 (0.80)	0.98 (0.72)	0.90 (0.62)	0.85 (0.62)
Soft drinks	1.14 (1.07)	1.19 (1.14)	1.15 (1.03)	1.10 (0.92)	0.98 (0.90)
Fruit juice	1.79 (1.35)	1.80 (1.41)	1.83 (1.29)	1.66 (1.09)	1.66 (1.29)
Other beverages	0.46 (0.49)	0.47 (0.52)	0.46 (0.46)	0.43 (0.39)	0.41 (0.42)
**Family functioning**	1.93 (0.43)	1.88 (0.42)	1.99 (0.44)	1.99 (0.46)	1.93 (0.41)
**Acculturation (AHIMSA scores)**					
^a^Integrated	3.56 (2.14)	5.47 (1.15)	1.60 (1.09)	1.32 (0.99)	3.03 (1.15)
^b^Assimilated	2.99 (1.96)	1.57 (1.02)	5.17 (1.18)	1.54 (0.94)	3.10 (1.08)
^c^Separated	1.19 (1.38)	0.78 (0.89)	0.98 (0.94)	4.46 (1.66)	1.58 (1.47)
^c^Marginalized	0.21 (0.57)	0.15 (0.43)	0.22 (0.51)	0.61 (1.15)	0.24 (0.64)
**Age group**					
8–12 years old	57.5%	46.5%	68.5%	60.9%	66.2%
13–16 years old	42.4%	53.5%	31.5%	39.1%	33.8%
**Sex**					
Female	49.6%	52.9%	47.6%	47.8%	49.5%
Male	50.3%	47.0%	52.3%	52.1%	50.4%
**Birthplace**					
U.S. Mainland	77.5%	73.2%	83.6%	71.9%	76.4%
Outside of the U.S. Mainland	22.4%	26.7%	15.3%	28.0%	22.0%
**Parental education**					
Less than high school	38.6%	39.3%	38.1%	47.8%	32.3%
High school/GED	28.5%	26.8%	29.4%	25.0%	32.8%
Four-yr college/university or more	32.8%	33.5%	32.1%	27.1%	34.3%
**Family income**					
Less than $20,000	52.9%	50.0%	51.0%	61.9%	49.0%
$20,000–$50,000	32.2%	32.9%	30.3%	26.0%	29.9%
Greater than $50,000	14.8%	13.7%	15.4%	09.7%	16.6%
**Household composition (number of members)**	4.28 (1.42)	4.29 (1.44)	4.24 (1.37)	4.38 (1.52)	4.30 (1.40)
**Acculturative stress**					
Acculturation conflicts	1.77 (0.78)	1.62 (0.69)	1.92 (0.82)	1.85 (0.79)	1.88 (0.84)
Perceived discrimination	1.47 (0.70)	1.34 (0.55)	1.57 (0.79)	1.75 (0.92)	1.50 (0.76)
Language conflicts	1.58 (0.86)	1.43 (0.72)	1.64 (0.92)	2.20 (1.05)	1.65 (0.88)
**Healthy Eating Index (2010-HEI)**	53.9 (12.8)	54.0 (13.0)	52.8 (12.6)	54.5 (12.7)	54.4 (12.5)

*Note*: ^a^Integration: Bi-culturalism; ^b^Assimilation: Anglo-dominant (U.S. orientation); ^c^Marginalization/Separation: Hispanic-dominant (Hispanic Orientation) or neither; ^d^Unclassified: Acculturation status unknown

Data shown as mean (SD) or percent

Family functioning score ranged from 1 to 4, with greater scores indicating poorer family functioning

AHIMSA score ranged from 1 to 8, with greater scores indicating greater acculturation for each category

GED: General Education Diploma

Acculturative Stress: The psychological impact of adaptation to a new culture. For Hispanics who come to the U.S., there are a number of significant stressors that are likely to be pervasive, intense, and lifelong due to acculturation. Each domain of the acculturative stress scale ranges from 1 to 5, with 5 more indicative of stress

Healthy Eating Index (2010-HEI) score ranges from 0 to 100 and is a measure of diet quality used to assess compliance with the 2010 U.S. Dietary Guidelines for Americans and monitor dietary changes; higher scores indicating greater consistency of the diet

Results from the path regression models are presented in Table 2. HEI and other demographic factors were excluded from models due to lack of preliminary correlation with key study variables (*r* < 0.20) and potential collinearity. Only youth’ age, sex, and study site were the key covariates included in our final models. When controlling for age, sex, and study site, acculturative stress (*β* = − 0.13, *p =* 0.01) was inversely associated with SSB among youth classified as assimilated, and poor family functioning (*β* = 0.11, *p =* 0.07) was only marginally associated with SSB consumption among youth classified as assimilated but not among youth classified as integrated, marginalized/separated, or unclassified.

**Table 2:** Adjusted multi-group (by acculturation status) path regression model of Hispanic/Latino youth’ SSB consumption by acculturative stress and family functioning (*n* = 1465)

Paths modeled	Integrated			Assimilated			Separated/Marginalized			Unclassified		
	β (SE)	95%CI	***p***	β (SE)	95%CI	***p***	β (SE)	95%CI	***p***	β (SE)	95%CI	***p***
**SSB intake < −- accult. Stress**	0.06 (0.06)	−0.06, 0.19	0.34	**−0.13 (0.04)**	**−0.22, −0.03**	**< 0.01**	0.65 (0.41)	−0.15, 1.45	0.11	− 0.18 (0.10)	− 0.38, 0.02	0.08
**SSB intake < −- poor family functioning**	− 0.00 (0.00)	− 0.01, 0.00	0.31	0.11 (0.06)	0.01, 0.23	0.07	−0.39 (0.45)	−1.28, 0.50	0.38	0.10 (0.06)	−0.02, 0.23	0.12
**Poor family functioning < −- accult. Stress**	**0.28 (0.09)**	**0.10, 0.46**	**< 0.01**	**0.55 (0.11)**	**0.32, 0.78**	**< 0.01**	**0.84 (0.39)**	**0.07, 1.61**	**0.03**	**0.29 (0.03)**	**0.22, 0.35**	**< 0.01**
**SSB intake < −- youth age**	**0.19 (0.03)**	**0.12, 0.25**	**< 0.01**	**0.23 (0.04)**	**0.13, 0.32**	**< 0.01**	**0.39 (0.16)**	**0.07, 0.71**	**0.01**	**0.17 (0.07)**	**0.03, 0.31**	**0.01**
**SSB intake < −- youth sex**	**0.07 (0.01)**	**0.03, 0.11**	**< 0.01**	**0.10 (0.04)**	**0.01, 0.20**	**0.02**	0.14 (0.17)	0.19, 0.48	0.39	0.08 (0.05)	−0.02, 0.20	0.12
**Poor family functioning < −- youth age**	0.07 (0.07)	− 0.07, 0.22	0.30	**0.22 (0.06)**	**0.09, 0.34**	**< 0.01**	0.15 (0.28)	−0.39, 0.70	0.57	0.06 (0.03)	−0.00, 0.13	0.08
**Poor family functioning < −- youth sex**	0.01 (0.03)	−0.06, 0.09	0.68	**−0.17 (0.04)**	**−0.26, − 0.08**	**< 0.01**	**−0.19 (0.09)**	**− 0.38, − 0.01**	**0.03**	**−0.09 (0.03)**	**− 0.16, 0.01**	**0.01**
**Accult. stress < −- youth age**	− 0.12 (0.11)	−0.35, 0.09	0.25	**−0.41 (0.05)**	**− 0.52, − 0.30**	**< 0.01**	**−0.56 (0.04)**	**− 0.64, − 0.45**	**< 0.01**	**−0.22 (0.09)**	**− 0.40, − 0.04**	**0.01**
**Accult. stress < −- youth sex**	**−0.17 (0.03)**	**− 0.24, − 0.10**	**< 0.01**	0.07 (0.05)	−0.03, 0.18	0.16	0.11 (0.17)	−0.22, 0.46	0.50	0.06 (0.05)	−0.05, 0.17	0.28
**R**^**2**^	0.09			0.29			0.49			0.10		
**Goodness-of-fit statistics**	**CFI = 0.99 | RMSEA = 0.02 (0.00, 0.05) | SRMR = 0.03**											

Abbreviations: *SSB* sugar-sweetened beverages; *Accult. Stress* acculturative stress; Acculturative stress is a latent variable composed of three observed indicators in the data, acculturative conflicts, perceived discrimination, and language conflicts (*a* = .61; *r* > .33). Indicators of acculturative stress loaded significantly onto factor (*b >* .41; *p* < 0.01)

Prior to testing the regression models, ANOVA tests indicated mean SSB intake was statistically different across acculturation groups (*F* (2,1301) = 3.43, *p* = 0.04). Chi-square tests also indicated statistical differences across groups in poor family functioning (X^2^ = 15.30, *p* < 0.01)

## Discussion

Findings from our study suggest limited support for our hypothesis that Hispanic/Latino youth’ SSB consumption differs by acculturation status and for the hypothesis that family functioning would be associated with increased SSB consumption among Hispanic/Latino youth across acculturating groups. Acculturation differed across Hispanic/Latino youth in our sample, but no significant associations were found between acculturation categories and SSB consumption. There was a significant inverse association between acculturative stress and increased SSB consumption among assimilated Hispanic/Latino youth, but not in other acculturating groups.

Previous research suggests higher rates of SSB consumption have been generally observed among low income and racial/ethnic minority children and adults, particularly Hispanics/Latinos, compared with their higher income and non-Hispanic white counterparts [15]. Past research also suggests that youth of Hispanic/Latino heritage experience greater risk of obesity-related outcomes, including diet [38, 39]. In our study, we found a significant relationship between acculturative stress and lower SSB consumption among youth classified as assimilated. On average, assimilated youth had greater acculturation conflicts compared to their counterparts with differing acculturation status. Whether related to diet or not, these conflicts could be further studied in their role limiting SSB consumption among assimilated youth. Another potential reason for this finding may be that assimilated Hispanic/Latino youth are more susceptible to being influenced by ‘diet culture.’ [40] Diet culture is a contemporary concept in which individuals adopt specific diets aiming at prioritizing or restricting consumption of foods based on broad societal standards. Future research should assess the implications for younger Hispanic/Latinos, who could be the most susceptible sub-group towards exposure to sport-based dietary marketing and potential restrictive eating patterns [41].

The Hispanic/Latino family is a vital context that shapes youth’ health behaviors [42–46]. In our study, poor family functioning was marginally associated with greater SSB consumption among assimilated Hispanic/Latino youth (not statistically significant). Additional research is needed to assess whether family (dys)function could be perceived as a stressor to assimilated Hispanic/Latino youth who may turn to unhealthy energy-balance behaviors as a coping mechanism [43]. Implications for older Latino youth who are assimilated to the U.S. should also be considered in future research within the context of this relationship, as they may be most susceptible to increased SSB consumption in poor functioning family contexts [39]. Should the family structure, through functioning and cohesion, shape beverage consumption among Hispanic/Latino children, families may need to build capacity and stronger ties to create more healthful home and family environments especially under challenging circumstances [27–29, 44].

This study has strengths worth mentioning. Sample size for the study population is representative of some Hispanic/Latino urban communities in the U.S with diverse countries of origin. In addition, validated measures were used to assess all key constructs in the analyses, including SSB consumption assessed with two 24-h recalls used to capture culturally-relevant beverages in this population as our study was collecting data [47]. Lastly, by accounting for acculturation as a grouping variable for assessing effect modification, rather than a predicting variable within the context of a cross-sectional study, we maximized the potential for examining acculturation in a more nuanced way in combination with other sociocultural determinants of health (i.e., acculturative stress, family functioning).

Limitations in the study include the cross-sectional and self-reported nature of the overall data, which could introduce error to the inferences made. Thus, we are unable to make causal inferences, but rather report on the cross-sectional links (or lack thereof) and encourage future research that can elucidate the potential causal pathways (i.e., mediating factors at the family, home, and policy levels). For instance, children’s SSB consumption may be influenced by acculturative factors through parents’ behaviors, practices, and their role shaping the home environment [48–51]. Also, we did not test for differences across Hispanic/Latino heritages due to small sample sizes, although studies among Hispanic/Latino adults have shown differences in acculturation and in SSB by heritage [21, 52]. Generalizability may be limited because some Hispanic/Latino youth may be underrepresented in relation to the geographic location of study recruitment. Lastly, future research is warranted to longitudinally assess acculturation as a process among Hispanic/Latino youth from diverse communities, as well as psychological and familial factors influencing links with dietary behaviors.

## Conclusion

In conclusion, there is an inverse association between acculturative stress and SSB consumption, as well as a marginal but insignificant association between family functioning and SSB consumption among assimilated Hispanic/Latino youth. These preliminary results may support further research to inform preventive family-based approaches to help improve diet quality among Hispanic/Latino children by dissuading SSB intake. Future studies should assess these important interconnections more rigorously and longitudinally, accounting for theoretically relevant covariates. Specifically, examining the interplay between key cultural (i.e., acculturation), familial, and behavioral (i.e., dietary behaviors) risk factors of obesity while accounting for racial and ethnic-specific characteristics could inform future obesity research and programming tailored to minority groups disproportionally affected by obesity.

## Data Availability

The datasets generated and/or analyzed during the current are available in the National Institutes of Health (NIH), National Heart, Lung, Blood Institute (NHLBI), Biologic Specimen and Data Repository Information Coordinating Center, [https://biolincc.nhlbi.nih.gov/studies/hchssol/].

## Abbreviations

SSB: Sugar-sweetened beverages
HCHS/SOL: Hispanic Community Children’s Health Study/Study of Latino
NDSR: Nutrition Data System for Research
AHIMSA: Acculturation, Habits, and Interests Multicultural Scale for Adolescents
HEI-2010: Healthy Eating Index
SEM: Structural Equation Modelling
